# Nebivolol, an FDA-Approved Drug, Has General Antibacterial and Antibiofilm Effects and Increases *Pseudomonas aeruginosa* Tolerance to Ciprofloxacin

**DOI:** 10.3390/ph17111472

**Published:** 2024-11-01

**Authors:** Yael Schlichter Kadosh, Noa Goorevitch, Kerem Teralı, Jacob Gopas, Ariel Kushmaro

**Affiliations:** 1 Avram and Stella Goldstein-Goren Department of Biotechnology Engineering, Ben Gurion University of the Negev, Beer Sheva 84105, Israel; shlicter@post.bgu.ac.il (Y.S.K.);; 2 Department of Medical Biochemistry, Faculty of Medicine, Cyprus Health and Social Sciences University, Morphou 99750, Cyprus; 3 Department of Microbiology, Immunology and Genetics Faculty of Health Sciences, Ben Gurion University of the Negev, Beer Sheva 84105, Israel; 4 The Ilse Katz Center for Nanoscale Science and Technology, Ben Gurion University of the Negev, Beer Sheva 84105, Israel; 5 School of Sustainability and Climate Change, Ben Gurion University of the Negev, Beer Sheva 84105, Israel

**Keywords:** nebivolol, FDA-approved drug, antibacterial resistance, *Pseudomonas aeruginosa*, biofilm

## Abstract

Background: The repurposing of approved drugs for new activities is gaining widespread attention, including drugs that have antibacterial properties. Nevertheless, besides the benefits of repurposing drugs, the discovery of new antibiotic activity in commonly used medicines raises concerns about inducing antibiotic tolerance and resistance due to the stress produced by the drugs. We found that nebivolol, which is used to treat hypertension, also has antibacterial activity. Methods: The antibacterial activity of nebivolol was tested by disc diffusion and kinetic O.D. measurements. Antibiofilm activity was determined by crystal violet staining. Results: Nebivolol has antibiotic and antibiofilm activity against several bacteria. However, its effect on *Pseudomonas aeruginosa*’s growth is limited, and it promotes biofilm formation. In addition, *P. aeruginosa* exposure to nebivolol induces resistance to ciprofloxacin but increases sensitivity to tobramycin. Conclusions: Nebivolol has antibiotic activity against several bacteria tested but is less effective and possibly detrimental in *P. aeruginosa* infections. The use of nebivolol, together with other antibiotics, should be further tested and carefully considered.

## 1. Introduction

Nebivolol is an FDA-approved drug for hypertension and related cardiovascular diseases. It enables heart relaxation by selectively blocking the beta 1 receptor in cardio-smooth muscles. Additionally, nebivolol increases the production of nitric oxide (NO), which causes vasodilation [1,2,3,4]. Nebivolol doses are prescribed daily between 2.5 and 20 mg in oral tablets once a day and it reaches peak plasma concentration after 1.5 to 4 h [5]. In 2016, nebivolol ranked 128 on the most-prescribed-drugs list, and 244 in 2021. Its use is decreasing due to the development of new classes of beta blockers [6].

The repurposing of approved drugs for new activities is gaining more attention due to regulatory advantages [7], including several drugs with antibacterial activity [8,9]. Nevertheless, besides the benefits of repurposing drugs, the discovery of new antibiotic activity in commonly used medicines raises concerns about inducing inheritable and noninheritable antibiotic tolerance and resistance due to the stress produced by the drugs [10]. These may cause difficulties in infection management. Nebivolol was previously found to have antibiotic activity toward *Acinetobacter baumannii*, with a Minimal Inhibitory Concentration (MIC) of above 100 µM (equal to ~40 µg/mL), which was the highest concentration tested. The implication of this result was not further discussed [11]. In this brief communication, we evaluated the antibiotic and antibiofilm activities of nebivolol and its effect on stress-induced bacterial resistance.

## 2. Results

**Figure 1:**
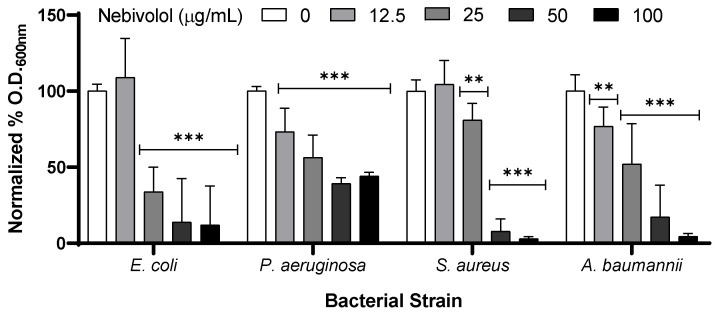
Antibacterial activity of nebivolol. *E. coli*, *P. aeruginosa*, *S. aureus*, and *A. baumannii* were incubated with increased concentrations of nebivolol in LB. Then, the 96-well plate was incubated at 37 °C and kinetically read at 600 nm. Reads were performed every 20 min overnight (16 h). The final read was normalized to the control and percentage. The graph presents the mean + SD, N = 9, in triplicates of three independent experiments. Statistical analysis was two-way ANOVA, followed by Dunnett’s multiple comparisons test (*p*-values: ** ≤ 0.0021, *** ≤ 0.0002), O.D = optical density.

The antibacterial activity of nebivolol was tested by disc diffusion and optical density (O.D) kinetics measurements. The zone of inhibition in *Escherichia coli* was 12 mm, that of *Staphylococcus aureus* was 12 mm, and that of *Acinetobacter baumannii* was 10 mm. Nebivolol was much less effective on *Pseudomonas aeruginosa* as no zone of inhibition was observed (0 mm) (Table 1). The Minimal Inhibitory Concentration (MIC) of nebivolol was 50 µg/mL for *E. coli*, *S. aureus*, and *A. baumannii*, and an MIC was not detected for *P. aeruginosa* (Figure 1).

**Figure 2:**
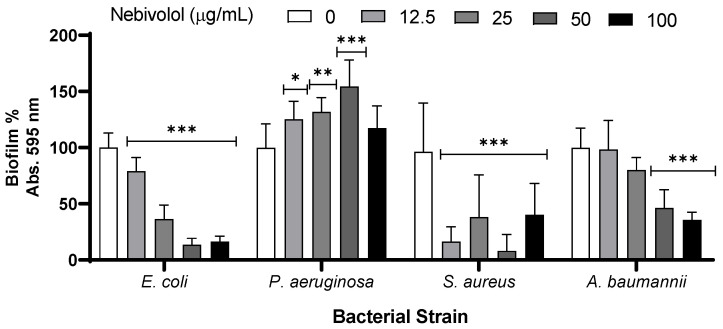
Effect of nebivolol on bacterial biofilm formation tested using crystal violet (CV) dye. *E. coli*, *P. aeruginosa*, *S. aureus*, and *A. baumannii* were incubated with increased concentrations of nebivolol in LB. The 96-well plate was incubated at 37 °C for 24 h at 37 °C, and the biofilm was dyed with 0.1% CV. The graph presents the subtraction of the blank, normalized against the control absorbance results, the percentage mean + SD, N = 9, in triplicates of three independent experiments. Statistical analysis was two-way ANOVA, followed by Dunnett’s multiple comparisons test (*p*-values: * ≤ 0.0332, ** ≤ 0.0021, *** ≤ 0.0002).

Biofilm formation is a refuge against different types of stressors, including noninheritable bacterial tolerance toward antibiotics [10,12]. Thus, we tested the effect of nebivolol on bacterial biofilm formation. *E. coli* and *A. baumannii* biofilms were sensitive to nebivolol in a dose-dependent manner. *S. aureus* biofilm was especially sensitive; however, no clear dose dependency was observed. In contrast, *P. aeruginosa* biofilm formation increased up to 50 µg/mL, and no significant difference was found between the biofilm of the untreated control and those treated with 100 µg/mL of nebivolol (Figure 2).

**Figure 3:**
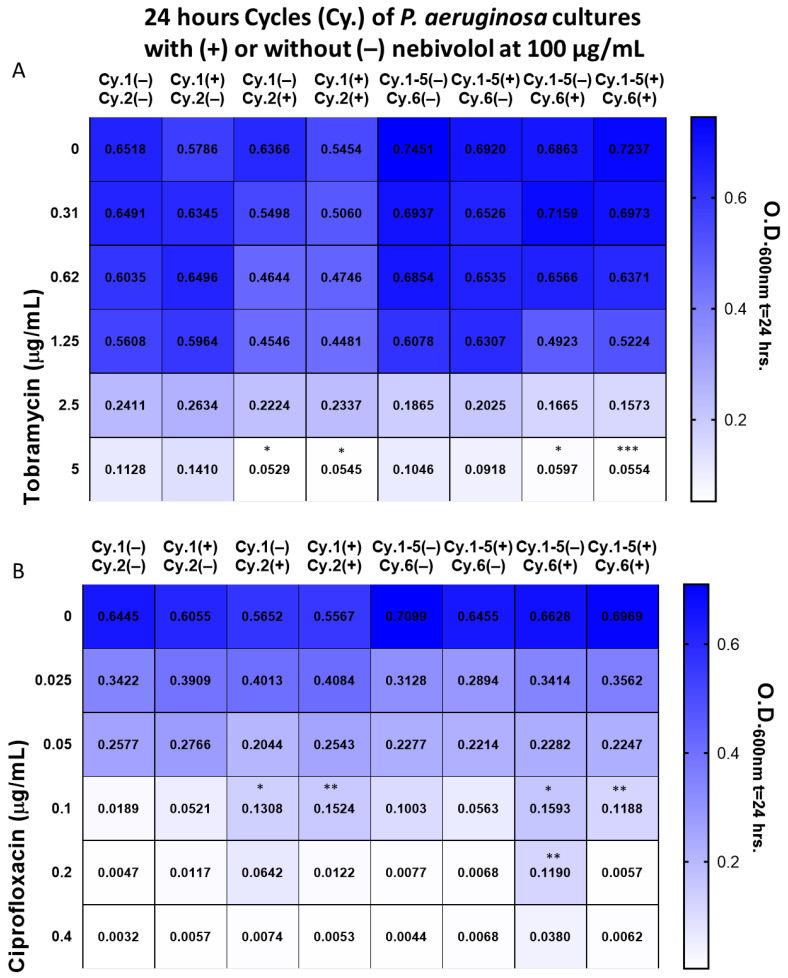
The effect of nebivolol pre-exposure and exposure on the response of *P. aeruginosa* to ciprofloxacin. The *P. aeruginosa* culture was diluted 1:100 in LB for five cycles; each cycle incubation time was 24 h at 37 °C, where the O.D. at 600 nm was read. From cycle 1 and cycle 5, the culture was transferred to challenged plates with increasing concentrations of (**A**) tobramycin or (**B**) ciprofloxacin, with or without nebivolol at 100 µg/mL. The figure shows the O.D. results of the challenged plates (cycles 2 and 6) after blank subtraction, and the mean of triplicates of three independent experiments (N = 9). Concentrations of 5 µg/mL of tobramycin and 0.1 and 0.2 µg/mL of ciprofloxacin were statistically tested via one-way ANOVA followed by Tukey’s multiple comparisons test (*p*-values * ≤ 0.0332, ** ≤ 0.0021, *** ≤ 0.0002). The stars represent the statistical significance of the treatment in comparison to the control in the same cycle.

Since nebivolol partially inhibits *P. aeruginosa* growth and increases biofilm formation, we also tested its effect on the antibiotic tolerance of the bacterium to the commonly used antibiotics tobramycin and ciprofloxacin. Pre-exposure and co-exposure to nebivolol showed a significant increase in sensitivity to tobramycin at 5 µg/mL (Figure 3A). The bacterial culture that was exposed to nebivolol from the beginning and throughout the experiment was significantly more resistant to ciprofloxacin than the control without nebivolol. When nebivolol and ciprofloxacin were added together to *P. aeruginosa* only at cycles 2 and 6, they became more resistant than the cultures that were exposed to nebivolol from the beginning and throughout the experiment (Figure 3B). Thus, nebivolol increases *P. aeruginosa*’s resistance to ciprofloxacin, but pre-exposure to it weakens the resistance.

## 3. Discussion

This work is focused on the implications of using FDA-approved drugs with unknown antibiotic activity on bacterial tolerance to known antibiotics. Drugs for the treatment of high blood pressure are frequently found at the top of the most-prescribed-drugs list [6]. Hypertension is one of the most common conditions globally [13,14]. Nebivolol is used to treat high blood pressure and heart diseases.

With the increasing interest in repurposing drugs for many new proposes, antibiotic activity should be used cautiously, and since antibiotics have negative effects on the microbiome [15,16,17], nebivolol should be further evaluated in this context. To the best of our knowledge, we are the first to report that nebivolol has antibiofilm activity against several bacterial strains, except for one article referring to the effect of nebivolol on *A. baumannii* biofilm. The authors showed that nebivolol was inactive, although they used a lower concentration (~20 µg/mL) than the concentration that we showed was active (50 and 100 µg/mL) on this bacterium [11].

Nebivolol moderately affects *P. aeruginosa* growth (in comparison to its effect on the other bacteria tested) and increases biofilm formation. Since bacteria that were exposed to sublethal doses of stressors showed increased resistance [18,19], we tested the effect of pre-exposure and co-exposure to nebivolol on the sensitivity of *P. aeruginosa* to tobramycin and ciprofloxacin. We found that while nebivolol does not significantly affect *P. aeruginosa*’s responses to tobramycin, the resistance to ciprofloxacin is considerably increased. That suggests that a patient who is treated with nebivolol can be treated with tobramycin but should avoid co-treatment with ciprofloxacin.

## 4. Materials and Methods

### 4.1. Bacterial Growth Conditions and Strains

The bacteria were struck on LB Miller agar Petri dishes from frozen glycerol stocks and incubated for 24 h at 37 °C. A few colonies were transferred to LB broth, incubated, and orbitally shaken (140 RPM) for 24 h at 37 °C. Then, the starter was diluted specifically for each experiment. The strains used in this study were *Acinetobacter baumannii* (ATCC No. 43498), *Escherichia coli* (ATCC No. 1100101), *Pseudomonas aeruginosa* PAO1 (ATCC No. 47085), *Staphylococcus aureus* (ATCC No. 33591), and *Bacillus subtilis* (ATCC No. 19659) [20].

### 4.2. Disc Diffusion Test of Nebivolol on Different Bacterial Strains

An amount of 10 µL of nebivolol (N1915, Sigma-Aldrich St. Louis, MO, USA) at 20 mg/mL (200 µg/disc) was placed on Wattman paper discs and dried. Then, the starters of *P. aeruginosa*, *S. aureus*, *A. baumannii*, and *E. coli* were evenly spread on LB agar plates, and the discs were placed on the cultures. Incubation was carried out for 24 h at 37 °C. The measurements of the zone of inhibition radius were determined.

### 4.3. Effect of Nebivolol on Bacterial Growth

To test the effect of nebivolol on bacterial growth, we kinetically measured O.D. 600 nm. The starters of *P. aeruginosa*, *S. aureus*, *A. baumannii*, and *E. coli* were diluted 1:100 in LB broth and transferred to 96-well plates with increasing concentrations of nebivolol (12.5, 25, 50, 100 µg/mL) or DMSO at equal concentrations. The measurements were performed in 20 min intervals overnight (16 h), with medium shaking, at 37 °C in a plate reader (Multiskan Go, Thermo Fisher Scientific, Madison, WI, USA). The O.D. of each sample at 16 h was normalized to that of the control.

### 4.4. The Effect of Nebivolol on Static Biofilm Formation

To test whether nebivolol affects the biofilm formation of *P. aeruginosa*, *S. aureus*, *A. baumannii*, and *E. coli*, their starters were diluted 1:100 in LB and divided into 96-well plates with increasing concentrations of nebivolol or DMSO. Then, the cultures incubated for 24 h. After, the liquid was discarded, and the biofilm was washed twice in phosphate-buffered saline (PBS) and dried completely in the air. The dried biofilm was dyed with 0.1% crystal violet for 20 min at room temperature (RT). The excess dye was discarded, and the wells were washed twice in PBS. To extract the dye, 95% ethanol was added for 1 h at RT. The solution was pipetted up and down thoroughly to confirm that the dye was completely extracted. Then, the extraction was transferred to a new plate and read in a plate reader at 595 nm. The blank absorbance was subtracted from the samples and normalized to the absorbance of the control.

### 4.5. Antibiotic Challenging of P. aeruginosa Exposed to Nebivolol

**Figure 4:**
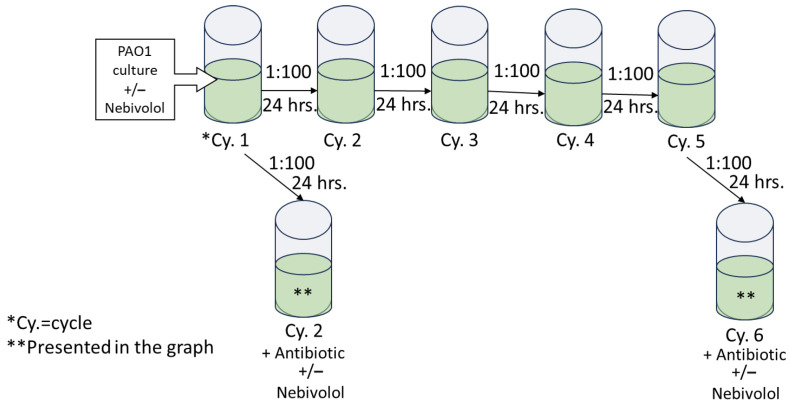
Illustration of antibiotic challenging of nebivolol-exposed *P. aeruginosa* culture. This *P. aeruginosa* culture was diluted 1:100 in LB for five cycles; each cycle incubation time was 24 h at 37 °C; and at the end, the O.D. at 600 nm was determined. From cycle 1 and cycle 5, the culture was transferred into challenged plates with increasing concentrations of antibiotics with or without nebivolol at 100 µg/mL.

*P. aeruginosa* starter was diluted 1:100 and these cultures were grown continuously with nebivolol for 6 cycles (pre-exposed). At cycles 2 and 6, ciprofloxacin or tobramycin was introduced, and the O.D. at 600 nm was determined after 24 h. In addition, *P. aeruginosa* cultures were grown in the absence of nebivolol for 6 cycles; at cycles 2 and 6, nebivolol and tobramycin or ciprofloxacin were added (co-exposure) for 24 h; and the O.D. at 600 nm was determined. Nebivolol concentration was 100 µg/mL, and the control contained DMSO at 0.5%. The bacteria were diluted in LB (1:100) for each cycle in a new 96-well plate, and incubations were carried out at 37 °C statically for 24 h. The protocol is illustrated in Figure 4.

### 4.6. Statistical Analysis

All statistical analyses were carried out using GraphPad Prism version 8.0.1 for Windows (GraphPad Software, v. 8.0.1., San Diego, CA, USA). The specific tests recommended by the software are indicated under the relevant figures.

## 5. Conclusions

To conclude, the literature shows that commonly used drugs for other conditions may also have antibiotic activity [21,22,23,24], as shown here for nebivolol. Although repurposing drugs may have great benefits, such as finding new antibiotic targets, research should be undertaken to investigate the possible development of damage to the microbiome and antibiotic resistance for the purpose of better clinical decision making. Moreover, nebivolol was found to inhibit the growth of *E. coli*, *S. aureus*, and *A. Baumannii* in a dose-dependent manner, suggesting the possibility of using nebivolol as a new antibiotic drug.

## Figures and Tables

**Table 1:** Nebivolol zone of inhibition. *E. coli*, *P. aeruginosa*, *S. aureus*, and *A. baumannii* overnight cultures were spread on LB agar plates, and then 200 µg of nebivolol or vehicle DMSO discs were loaded on top of them and incubated at 37 °C for 24 h. The zone of inhibition was measured.

Bacterial Strain	Zone of Inhibition Image Nebivolol	Zone of Inhibition Image DMSO	Diameter of Inhibition (mm)
*Bacillus sabtilis*	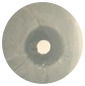	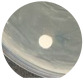	18
*Staphylococcus aureus*	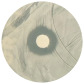	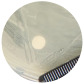	12
*Pseudomonas aeruginosa*	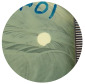	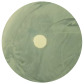	0
*Escherichia coli*	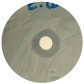	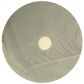	12
*Acinetobacter baumannii*	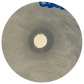	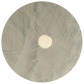	10

## Data Availability

Data are available from the corresponding author upon request.
